# The fixed-effects model for robust analysis of stepped-wedge cluster trials with a small number of clusters and continuous outcomes: a simulation study

**DOI:** 10.1186/s13063-024-08572-1

**Published:** 2024-10-25

**Authors:** Kenneth Menglin Lee, Yin Bun Cheung

**Affiliations:** 1 Centre for Quantitative Medicine, Duke-NUS Medical School, 8 College Road, Singapore, 169857 Singapore; 2 Signature Research Programme in Health Services & Systems Research, Duke-NUS Medical School, Singapore, 169857 Singapore; 3 Tampere Center for Child, Adolescent and Maternal Health Research, Tampere University, Tampere, 33520 Finland

**Keywords:** Stepped wedge, Cluster trials, Simulation study, Fixed effects

## Abstract

**Background:**

Stepped-wedge cluster trials (SW-CTs) describe a cluster trial design where treatment rollout is staggered over the course of the trial. Clusters are commonly randomized to receive treatment beginning at different time points in this study design (commonly referred to as a Stepped-wedge cluster randomized trial; SW-CRT), but they can also be non-randomized. Trials with this design regularly have a low number of clusters and can be vulnerable to covariate imbalance. To address such covariate imbalance, previous work has examined covariate-constrained randomization and analysis adjustment for imbalanced covariates in mixed-effects models. These methods require the imbalanced covariate to always be known and measured. In contrast, the fixed-effects model automatically adjusts for all imbalanced time-invariant covariates, both measured and unmeasured, and has been implicated to have proper type I error control in SW-CTs with a small number of clusters and binary outcomes.

**Methods:**

We present a simulation study comparing the performance of the fixed-effects model against the mixed-effects model in randomized and non-randomized SW-CTs with small numbers of clusters and continuous outcomes. Additionally, we compare these models in scenarios with cluster-level covariate imbalances or confounding.

**Results:**

We found that the mixed-effects model can have low coverage probabilities and inflated type I error rates in SW-CTs with continuous outcomes, especially with a small number of clusters or when the ICC is low. Furthermore, mixed-effects models with a Satterthwaite or Kenward-Roger small sample correction can still result in inflated or overly conservative type I error rates, respectively. In contrast, the fixed-effects model consistently produced the target level of coverage probability and type I error rates without dramatically compromising power. Furthermore, the fixed-effects model was able to automatically account for all time-invariant cluster-level covariate imbalances and confounding to robustly yield unbiased estimates.

**Conclusions:**

We recommend the fixed-effects model for robust analysis of SW-CTs with a small number of clusters and continuous outcomes, due to its proper type I error control and ability to automatically adjust for all potential imbalanced time-invariant cluster-level covariates and confounders.

**Supplementary Information:**

The online version contains supplementary material available at 10.1186/s13063-024-08572-1.

## Background

The cluster randomized trial (CRT) is a useful alternative to the individually randomized controlled trial where clusters of individuals, rather than the individuals themselves, are randomized to receive the intervention [1, 2]. CRTs are often used when the intervention needs to be administered at the cluster-level or when there is a risk of contamination between intervention groups.

CRTs with a low number of randomized clusters can be vulnerable to covariate imbalance. Such covariate imbalance can lead to biased intervention effect estimates, inflated standard errors, and reduced power for the intervention effect in both parallel cluster randomized trials (P-CRTs) [3] and stepped-wedge cluster randomized trials (SW-CRTs) [4]. In this article, we will focus on methods to address cluster-level, between-sequence, baseline covariate imbalance in stepped-wedge designs [5].

Both P-CRTs and SW-CRTs are often analyzed with a mixed-effects model, which specifies cluster intercepts as random effects [6]. The mixed-effects model relies on randomization to control for known and unknown confounders. However, the benefits of randomization may be lost when the number of clusters is small [7, 8]. Due to real-world constraints, it is not uncommon for SW-CRTs to have low numbers of clusters [5, 9]; in 2016, Barker et al. reported around 20% of SW-CRT studies had 5 or fewer clusters [9]. Previous papers have examined covariate-constrained randomization and or adjustment for imbalanced covariates in mixed-effects models of P-CRTs [3, 10–12] and SW-CRTs [4, 5, 13] to alleviate the negative influences of covariate imbalance. However, these methods require the imbalanced covariate to always be known and measured, which may not always be practical. For example, in SW-CRTs where clusters are hospital wards, patient attribute data is often collected. However, data on the attributes (such as attitude, abilities, and general practice habits) of clinicians administering the intervention may not always be collected and can lead to an imbalance in patient outcomes between clusters [14].

Furthermore, beyond cluster-level covariate imbalance, there may also be scenarios in stepped-wedge designs where cluster-level confounding exists. In practice, non-randomized stepped-wedge trials have been implemented to evaluate the effects of a multi-component knowledge translation intervention [15], a mammography intervention [16], evidence-based quality improvement interventions [17], and a prospective frontline surveillance system [18]. To clarify, in such scenarios, it may be a misnomer to refer to such trials as “stepped-wedge cluster randomized trials” as randomization is not implemented. From here on, we will broadly refer to these designs (both randomized and non-randomized) as “stepped-wedge cluster trials” (SW-CTs), with SW-CRTs specifically referring to SW-CTs with cluster randomization.

As an alternative to the mixed-effects model, we can instead specify cluster intercepts as fixed dummy variables using a fixed-effects model to analyze data collected from a SW-CT [19, 20]. Previous SW-CT studies have used the fixed-effects model, citing difficulties that arise from a small number of clusters [21, 22], practical and logistical issues that prevented randomization [15], and concerns over confounding between clusters and outcomes [23]. Fixed-effects models are commonly understood to “make within-unit comparisons” and adjust for all unit-level covariates and confounding [24, 25]. In this context with clustered trial designs, these “units” specifically refer to the clusters. Accordingly, in the analysis of SW-CTs, the fixed-effects model is expected to automatically adjust for all imbalanced time-invariant cluster-level covariates, both measured and unmeasured. However, previous work on covariate imbalance in SW-CRTs has neglected to evaluate the fixed-effects model as a potential alternative method [4, 5, 13]. Furthermore, the extent to which the fixed-effects model can control for cluster-level confounding in SW-CTs, as compared to mixed-effects models, has not been explicitly demonstrated.

Furthermore, the mixed-effects model can have inflated type I error rates and overly narrow confidence intervals in P-CRTs with a small number of clusters [26, 27]. Notably, these issues can be largely resolved in mixed-effects model analyses of P-CRTs by including a Satterthwaite or Kenward–Roger small sample correction [27]. We will explore how these small sample corrections perform in the mixed-effects model analyses of SW-CTs.

Similar inflated type I errors have been previously observed when using the mixed-effects model in the analysis of SW-CTs with a small number of clusters and binary outcomes [28]. This inflated type I error rate was not observed in the fixed-effects model analysis of SW-CTs with binary outcomes, making it an attractive alternative [28]. However, these results have not yet been extended to SW-CTs with continuous outcomes, nor has the fixed-effects model been compared to the mixed-effects model with small sample corrections in the context of SW-CTs. Fixed-effects models using unit-level (in this case cluster-level) dummy variables have been previously demonstrated to differ in properties between analyses with binary or continuous outcomes [24]. Famously, Robinson and Jewell demonstrated that the precision gain resulting from covariate adjustment differed between analyses with continuous outcomes (using a linear link function) and with binary outcomes (using a logistic link function) [29]. Accordingly, we aim to fill gaps in the literature regarding the application of the fixed-effects model to SW-CTs with continuous outcomes.

Overall, the use of the fixed-effects model is a departure from the status quo in the analysis of SW-CTs and additional considerations must be made when using it. Broadly, the standard mixed-effects model induces an exchangeable correlation structure by including a cluster random intercept in the analysis [6]. Unlike corresponding analyses but excluding the cluster random intercept (sometimes referred to as an independence estimating Eq. [30, 31]), this cluster random intercept in a mixed-effects model adjusts for the between-cluster variance which is equivalent to the covariance between observations within the same cluster [6, 24]. Unlike an independence estimating equation, the fixed-effects model adjusts for the covariance between observations within the same cluster by capturing it in the cluster fixed effect terms [24]. Subsequently, observations within clusters are considered conditionally independent in a fixed-effects model after conditioning on a given cluster fixed intercept, as is also the case in a mixed-effects model after conditioning on a given cluster random intercept (Additional File 1.i). Indeed, Mundlak [32] made the argument that the fixed-effects model can be interpreted as the more generalized form of the mixed-effects model with fewer model assumptions [24, 32, 33].

Since the between-cluster variance is explicitly controlled for by the cluster fixed intercepts, the fixed-effects model does not automatically provide an estimate of the between-cluster variance (like the mixed-effects model) which can complicate the estimation of the intracluster correlation (ICC) [34]. Notably, the CONSORT extension to SW-CRTs suggests including estimates of the ICC when reporting results to help inform future studies [35]. Accordingly, we will describe alternative ways to estimate the ICC, even while using a fixed-effects model, by estimating the ICC in a separate step from the analysis of the treatment effect.

In this article, we present a simulation study to compare the performance of the fixed-effects model against the mixed-effects model in the analyses of cross-sectional SW-CTs with small numbers of clusters, continuous outcomes, cluster-level covariate imbalances, and cluster-level confounding. In doing so, we aim to address notable gaps in the SW-CT literature (i.) concerning the performance of the fixed-effects model in controlling for inflated type I error rates in trials with continuous outcomes and (ii.) by explicitly demonstrating the fixed-effects model’s ability to automatically adjust for all time-invariant cluster-level covariate imbalance and confounding. Additionally, we address misconceptions regarding the inferential target for the mixed-effects and fixed-effects models. In Section "Methods", we describe the simulation data-generating process (DGP), analytic models of interest, and the simulation scenarios. In Section "Results", we present and discuss the simulation results. In Sections "Discussion" and "Conclusion", we end with some concluding remarks.

## Methods

### Data simulation

We will simulate cross-sectional SW-CT data with continuous outcomes, an exchangeable correlation structure, and an additional time-invariant binary covariate [3, 4, 36]:

Equation 1

$${Y}_{ijk}={\phi }_{j}+X_{ij}\delta +{C}_{i}\xi +{\alpha }_{i}+{e}_{ijk}$$

$${\alpha }_{i}\stackrel{iid}{\sim }N\left(0, {\tau }_{\alpha }^{2}\right)$$

$${e}_{ijk}\stackrel{iid}{\sim }N(0,{\sigma }_{w}^{2})$$

where $Y_{ijk}$ is the continuous outcome for individual $k$ $(k=1,...,n_{ij})$ in cluster $i$ $(i=1,\dots ,I)$ at period $j$ $(j=1,\dots ,J)$. ${\phi }_{j}$ is the $J$ fixed effects for each period. ${X}_{ij}$ is the indicator for the intervention effect $\delta$ in each cluster-period cell. We also include an additional time-invariant cluster-level binary covariate ${C}_{i}$ with covariate effect $\xi$. ${\alpha }_{i}$ is the normally distributed random intercept for the $i^{th}$ cluster, representing the culminative effect of all unmeasured cluster-level covariates. ${e}_{ijk}$ is the residual independent of ${\alpha }_{i}$. The cluster random intercept ${\alpha }_{i}$ accordingly induces an intracluster correlation coefficient $\left(\text{ICC}=\frac{{\tau }_{\alpha }^{2}}{{\tau }_{\alpha }^{2}+{\sigma }_{w}^{2}}\right)$ between individual outcomes within the same cluster.

### Simulation scenarios

**Fig. 1:**
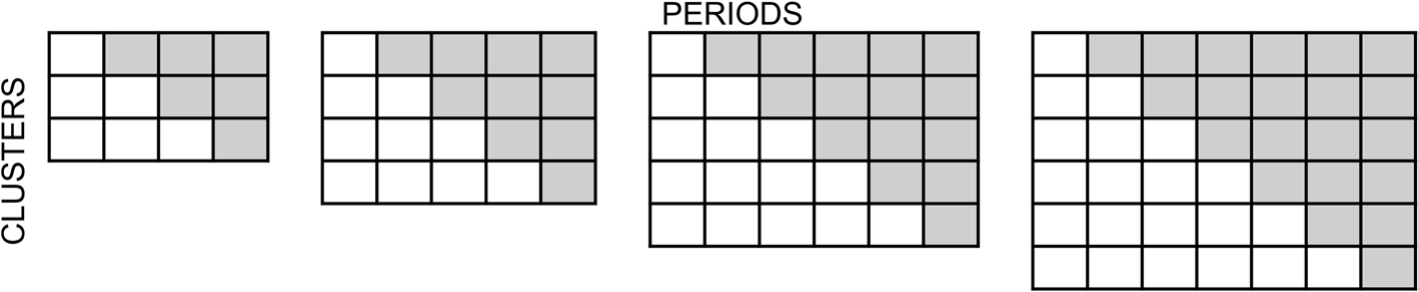
SW-CT designs with three, four, five, and six sequences with one cluster in each sequence

In 2016, Barker et al. reported around 20% of SW-CT studies had five or fewer clusters [9]. A 2023 review of published SW-CT studies by Nevins et al. reported that the median number of cross-over sequences was 5 with the majority of studies randomizing 1 cluster to each sequence [5]. Accordingly, we simulated small SW-CT designs with 3, 4, 5, 6 and 10 sequences, with 1 cluster in each sequence (Fig. 1).

In each of these scenarios, we used a range of different average cluster-period sample sizes ${\overline{n} }_{i}$ across the $J$ periods in the ${i}^{\text{th}}$ cluster, where ${\overline{n} }_{i}\sim \text{Gamma}\left(k,\theta \right)$ with $k=$ $30$, $100$, and $\theta =1$, producing an average cluster-period sample size of $30$ or $100$. Realized sample sizes ${n}_{ij}$ for the ${i}^{\text{th}}$ cluster during the ${j}^{\text{th}}$ period were subsequently generated with ${n}_{ij}\sim \text{Poisson}\left({\overline{n} }_{i}\right)$, so the sample size could vary between periods within a cluster. The trial’s total sample size was $N={\sum }_{i}{\sum }_{j}{n}_{ij}$.

We generated residual error ${e}_{ijk}$ by setting ${e}_{ijk}\sim \text{Normal}\left(0, {\sigma }_{w}^{2}=1\right)$. To simulate cluster effects, we set the between-cluster variance ${\tau }_{\alpha }^{2}$ to $1/99$, $1/19$, $1/9,$ and $1/4$, to generate corresponding intracluster correlation coefficient (ICC) values of $0.01$, $0.05$, $0.1,$ and $0.2$, where ICC $=\frac{ {\tau }_{\alpha }^{2}}{ {\tau }_{\alpha }^{2}+{\sigma }_{w}^{2}}$ [6].

We generated the true intervention effect $\delta$ equal to $0$ for the null effect and $0.5$ for a medium effect size. We also generated a linear period effect equal to an increase of $0.2$ per period for a small effect size. We included a cluster-level covariate effect of $\xi$ equal to 0 and 0.3.

The first scenario (i.) involves SW-CTs with no cluster-level covariate imbalance or confounding. To clarify, such SW-CT scenarios are examples of SW-CRTs, due to the randomization being preserved. Similarly, scenarios where SW-CTs are (ii.) simulated with chance cluster-level covariate imbalance can also be accurately referred to as SW-CRTs. Additionally, we describe two more SW-CT simulation scenarios with (iii.) cluster-level covariate confounding or (iv.) complete cluster-level confounding, where randomization may not occur.

#### Simulating time-invariant cluster-level chance covariate imbalance

In both scenarios (i.) and (ii.), we simulated scenarios with time-invariant cluster-level chance covariate imbalance where ${C}_{i}\sim \text{Bernoulli}\left(0.5\right)$. With such cluster-level covariate imbalance, the binary covariate ${C}_{i}$ is independent of the sequence allocation and the assigned treatment status during a given period. We set $\xi =0$ and $\xi =0.3$ in scenarios (i.) and (ii.), respectively (Eq. 1), such that the data generating process in scenario (i.) has no cluster-level covariate imbalance or confounding and scenario (ii.) has chance cluster-level covariate imbalance.

In scenario (ii.), we additionally examined the bias produced by different model estimators given different levels of chance covariate imbalance severity, similar to a previously published simulation study of covariate imbalance in P-CRTs [3]. We illustrate the different biases across these different imbalance levels in an additional batch of simulations with ICC values of $0.01$, $0.05$, $0.1$, $0.2$, and average cluster-period sample sizes of 100 individuals in scenarios with 3 clusters randomized to 3 sequences.

We use this simple three-cluster, four-period SW-CT design to simply define three different quantiles of covariate imbalance severity. Where the covariate imbalance indicators are $\left({C}_{1},{C}_{2},{C}_{3}\right)$ with ${C}_{i}=0$ or $1$ for clusters $i=\text{1,2},3$, respectively, we can define the high negative imbalanced quantile Q1: $\left[\left(\text{0,0},1\right),\left(\text{0,1},1\right)\right]$, balanced quantile Q2: $\left[\left(\text{0,1},0\right),\left(\text{1,0},1\right)\right]$, and high positive imbalanced quantile Q3: $\left[\left(\text{1,0},0\right),\left(\text{1,1},0\right)\right]$, excluding conditions where all clusters do not have the imbalanced covariate $\left({C}_{i}=0 \forall i\right)$ or have the imbalanced covariate $\left({C}_{i}=1 \forall i\right)$. In the high negative (Q1) or positive (Q3) imbalanced quantiles, the clusters with the imbalanced covariate are randomized to receive the treatment either later or earlier in the trial, respectively, which we hypothesize will lead to biased treatment effect estimates within these quantiles based on previous work in P-CRTs [3].

#### Simulating time-invariant cluster-level covariate confounding

In scenario (iii.), we additionally simulated scenarios with time-invariant cluster-level covariate confounding by simply generating the cluster-level covariates, setting $\xi =0.3$, and ordering the clusters such that the clusters receiving the treatment in later periods have the imbalanced covariate. This resembles simulating clusters from exclusively a high negative imbalanced quantile as described in Section "Simulating time-invariant cluster-level chance covariate imbalance". With such covariate confounding, the binary covariate ${C}_{i}$ is correlated with the sequence allocation and subsequently the assigned treatment status during a given period.

#### Simulating complete cluster-level confounding

We also simulated scenarios with (iv.) complete cluster-level time-invariant confounding by ordering clusters $i$ by their corresponding ${\alpha }_{i}$ values and assigning clusters that have lower ${\alpha }_{i}$ values to sequences that crossover to the intervention condition at earlier periods. Such extreme confounding may occur in practice following data collection in the baseline period of a SW-CT, where clusters with lower baselines are selected to begin receiving the intervention at earlier time points.

Following the sorting of clusters by their corresponding ${\alpha }_{i}$ values, we additionally generate a confounding cluster-level covariate that is ordered such that clusters receiving the treatment in later periods have the covariate. However, we set the covariate to have a null effect $\xi =0$, allowing it to instead serve as an imperfect proxy indicator for the complete cluster-level confounding.

### Analytic models of interest

#### Mixed-effects model

We can analyze the intervention effects in a SW-CT using the “Hussey & Hughes” mixed-effects model [6] with a cluster random effect as specified in Eq. 1 with $\xi =0$, shown below as Eq. 2.1:

Equation 2.1

$$Y_{ijk}=\phi_j+X_{ij}\delta+a_i+e_{ijk}$$

$$a_i\overset{iid}\sim N(0,\tau_a^2)$$

$$e_{ijk}\overset{iid}\sim N(0,\sigma_w^2).$$

Throughout this article, we will generally refer to this model (Eq. 2.1) simply as the mixed-effects model.

The intervention effect $\delta$ can then be estimated using weighted least squares (WLS). Equation 2.1 can be rewritten as:

$${Y}_{ijk}={\dot{Z}}_{ijk}\dot{\theta }+{\epsilon }_{ijk}$$

$${\epsilon }_{ijk}\sim N(0,{V}_{ijk})$$

with ${Y}_{ijk}$ being the $({\sum }_{i=1}^{I}{\sum }_{j=1}^{J}{n}_{ij})$ by $1$ vector individual level outcomes [6]. ${\dot{Z}}_{ijk}$ is the conventional $({\sum }_{i=1}^{I}{\sum }_{j=1}^{J}{n}_{ij})$ by $\left(J+1\right)$ design matrix and $\dot{\theta }$ is the $(J+1)$ by 1 vector of parameters, $({\phi }_{1},\dots ,{\phi }_{J},\delta )'$. ${V}_{ijk}$ denotes the variance–covariance matrix of ${Y}_{ijk}$ and is an $({\sum }_{i=1}^{I}{\sum }_{j=1}^{J}{n}_{ij})$ by $({\sum }_{i=1}^{I}{\sum }_{j=1}^{J}{n}_{ij})$ block diagonal matrix. ${V}_{ijk}{=\text{I}}_{I}\otimes {R}_{i}$ (where ${\text{I}}_{I}$ is an $I$ by $I$ dimension identity matrix):

$${V}_{ijk}=\left(\begin{array}{cccc}{R}_{1}& 0& \cdots & 0\\ 0& {R}_{i}& \cdots & 0\\ \vdots & \vdots & \ddots & \vdots \\ 0& 0& \cdots & {R}_{I}\end{array}\right)$$

and each block ${R}_{i}$ is a ${\sum }_{j=1}^{J}{n}_{ij}$ by ${\sum }_{j=1}^{J}{n}_{ij}$ symmetric matrix:

$${R}_{i}={\text{I}}_{{\sum }_{j=1}^{J}{n}_{ij}}{\sigma }_{w}^{2}+{\text{J}}_{{\sum }_{j=1}^{J}{n}_{ij}}{\tau }_{\alpha }^{2}=\left(\begin{array}{cccc}{\sigma }_{w}^{2}+{\tau }_{\alpha }^{2}& {\tau }_{\alpha }^{2}& \cdots & {\tau }_{\alpha }^{2}\\ {\tau }_{\alpha }^{2}& {\sigma }_{w}^{2}+{\tau }_{\alpha }^{2}& \cdots & {\tau }_{\alpha }^{2}\\ \vdots & \vdots & \ddots & \vdots \\ {\tau }_{\alpha }^{2}& {\tau }_{\alpha }^{2}& \cdots & {\sigma }_{w}^{2}+{\tau }_{\alpha }^{2}\end{array}\right).$$

where ${\text{I}}_{{\sum }_{j=1}^{J}{n}_{ij}}$ is a ${\sum }_{j=1}^{J}{n}_{ij}$ by ${\sum }_{j=1}^{J}{n}_{ij}$ dimension identity matrix and ${\text{J}}_{{\sum }_{j=1}^{J}{n}_{ij}}$ is a ${\sum }_{j=1}^{J}{n}_{ij}$ by ${\sum }_{j=1}^{J}{n}_{ij}$ dimension matrix of ones.

The resulting parameter point and variance estimators are then ${\left({\dot{Z}}_{ijk}'{V}_{ijk}^{-1}{\dot{Z}}_{ijk}\right)}^{-1}{\dot{Z}}_{ijk}'{V}_{ijk}^{-1}{Y}_{ijk}$ and ${\left({\dot{Z}}_{ijk}'{V}_{ijk}^{-1}{\dot{Z}}_{ijk}\right)}^{-1}$, respectively.

Additionally, we analyze the data with a covariate-adjusted mixed-effects model, shown below as Eq. 2.2:

Equation 2.2

$${Y}_{ijk}={\phi }_{j}+{X}_{ij}\delta +{C}_{i}\xi +{\alpha }_{i}+{e}_{ijk}$$

$${\alpha }_{i}\stackrel{iid}{\sim }N\left(0, {\tau }_{\alpha }^{2}\right)$$

$${e}_{ijk}\stackrel{iid}{\sim }N(0,{\sigma }_{w}^{2})$$

where we include a dummy variable ${C}_{i}$ to adjust for the time-invariant cluster-level binary covariate effect $\xi$. Equation 2.2 can then be rewritten as:

$${Y}_{ijk}={\ddot{Z}}_{ijk}\ddot{\theta }+{\epsilon }_{ijk}$$

$${\epsilon }_{ijk}\stackrel{iid}{\sim }N(0,{V}_{ijk})$$

where ${\ddot{Z}}_{ijk}$ is the conventional $({\sum }_{i=1}^{I}{\sum }_{j=1}^{J}{n}_{ij})$ by $\left(J+2\right)$ design matrix and $\ddot{\theta }$ is the $(J+2)$ by 1 vector of parameters, $({\phi }_{1},\dots ,{\phi }_{J},\delta ,\xi )'$.

#### Fixed-effects model

As an alternative to the mixed-effects model, we can instead model cluster intercepts as fixed dummy variables using the fixed-effects model, shown below as Eq. 3:

Equation 3

$${Y}_{ijk}={\phi }_{j}+{X}_{ij}\delta +{\alpha }_{i}+{e}_{ijk}$$

$${e}_{ijk}\stackrel{iid}{\sim }N(0,{\sigma }_{w}^{2})$$

where ${\phi }_{j}$ are the $J-1$ fixed effects for each period and ${\alpha }_{i}$ are the $I$ fixed effects for each cluster. The cluster fixed intercepts ${\alpha }_{i}$ automatically control for all measured and unmeasured cluster-level time-invariant confounders [24], including the cluster-level time-invariant imbalanced covariate ${C}_{i}$ with effect $\xi$.

The intervention effect in the fixed-effects model can then be estimated using ordinary least squares (OLS). Equation 3 can be rewritten as:

$${Y}_{ijk}={\widetilde{Z}}_{ijk}\widetilde{\theta }+{\widetilde{\epsilon }}_{ijk}$$

$${\widetilde{\epsilon }}_{ijk}\stackrel{iid}{\sim }N(0,{\widetilde{V}}_{ijk})$$

with ${Y}_{ijk}$ being the $({\sum }_{i=1}^{I}{\sum }_{j=1}^{J}{n}_{ij})$ by $1$ vector individual level outcomes. ${\widetilde{Z}}_{ijk}$ is the conventional $({\sum }_{i=1}^{I}{\sum }_{j=1}^{J}{n}_{ij})$ by $\left(J+I\right)$ design matrix and $\widetilde{\theta }$ is the $(J+I)$ by 1 vector of parameters, $({\phi }_{2},\dots ,{\phi }_{J},\delta ,{\alpha }_{1},\dots ,{\alpha }_{I})'$. ${\widetilde{V}}_{ijk}{=\text{I}}_{{\sum }_{i=1}^{I}{\sum }_{j=1}^{J}{n}_{ij}}{\sigma }_{w}^{2}$ (where ${\text{I}}_{{\sum }_{i=1}^{I}{\sum }_{j=1}^{J}{n}_{ij}}$ is an ${\sum }_{i=1}^{I}{\sum }_{j=1}^{J}{n}_{ij}$ by ${\sum }_{i=1}^{I}{\sum }_{j=1}^{J}{n}_{ij}$ dimension identity matrix) denotes the variance–covariance matrix of ${Y}_{ijk}$.

The resulting parameter point and variance estimators are then ${\left({\widetilde{Z}}_{ijk}'{\widetilde{Z}}_{ijk}\right)}^{-1}{\widetilde{Z}}_{ijk}'{Y}_{ijk}$ and ${\sigma }_{w}^{2}{\left({\widetilde{Z}}_{ijk}'{\widetilde{Z}}_{ijk}\right)}^{-1}$, respectively.

#### Alternative approaches to ICC estimation in a SW-CT

Notably, the mixed-effects model automatically estimates the variance of the cluster random intercepts, which can be subsequently used to derive the intracluster correlation coefficient (ICC). In contrast, the fixed-effects model does not automatically estimate the ICC in the analysis of a SW-CT. Accordingly, we propose estimating the ICC in a separate step from the analysis of the treatment effect. We discuss three different approaches below.

The ICC can be estimated by only using data from the unexposed first period $j=1$ in the SW-CT design with the following random effects model:

$${Y}_{i1k}=\mu +{\alpha }_{i}+{e}_{i1k}$$

$${\alpha }_{i}\stackrel{iid}{\sim }N\left(0, {\tau }_{\alpha }^{2}\right)$$

$${e}_{i1k}\stackrel{iid}{\sim }N\left(0,{\sigma }_{w}^{2}\right)$$

where $\mu$ is specified here as the average of the cluster effects and ${\alpha }_{i}$ is the normally distributed cluster random effect for the ${i}^{\text{th}}$ cluster.

Alternatively, the ICC can be estimated only using data from the unexposed first period and always-exposed final period in the SW-CT design with the following mixed-effects model:

$${Y}_{ijk}={\phi }_{j}+{\alpha }_{i}+{e}_{ijk}$$

$${\alpha }_{i}\stackrel{iid}{\sim }N\left(0, {\tau }_{\alpha }^{2}\right)$$

$${e}_{ijk}\stackrel{iid}{\sim }N\left(0,{\sigma }_{w}^{2}\right)$$

where ${\phi }_{j}$ are the 2 fixed effects for periods $j=1$ and $J$, and ${\alpha }_{i}$ is the normally distributed cluster random effect for the ${i}^{th}$ cluster.

Finally, the ICC can also be estimated using the previously described mixed-effects model (Eq. 2.1).

We will validate the performance of these approaches in the simulation scenarios with (i.) no cluster-level covariate imbalance or confounding (Section "No cluster-level covariate imbalance or confounding") and (iv.) complete cluster-level time-invariant confounding (Additional File 1.ii).

#### Inference target of mixed-effects and fixed-effects models

Historically, the mixed-effects and fixed-effects models have been mistakenly interpreted as each innately estimating coefficients over the entire superpopulation of all possible clusters or only the finite population of sampled clusters, respectively [37]. In contrast, it has long been argued in the econometrics literature that the choice of whether cluster intercepts are modeled as random or fixed is distinct from the inference space [32]. We speculate that much of the confusion over the inference space of mixed-effects and fixed-effects models may arise from the fact that the mixed-effects model directly models the presumed underlying data-generating process (DGP) that assumes clusters are randomly sampled. Indeed, under such a scenario with a correctly specified correlation structure, the mixed-effects model is the best linear unbiased estimator (BLUE) [32]. However, to clarify, it is the cluster random sampling itself that allows for the extrapolation of results from a given trial and its sampled clusters to the larger superpopulation of clusters [38], not the chosen analytic model.

The nomenclature referring to cluster intercepts as “random” or “fixed” further adds to the confusion, as previous publications have pointed out [32, 39]. Cluster effects can be assumed random and still analyzed using a fixed-effects model, with the important distinction between mixed-effects and fixed-effects models being whether the cluster effects are correlated with the other model covariates, and hence confounding [32, 34, 39]. Accordingly, both the mixed-effects model and fixed-effects model can be applied under either a superpopulation or finite sample framework [30, 31, 40].

This discussion is further complicated by the ambiguous language often used when discussing conditional and marginal models, which are commonly referred to as “subject-specific” and “population-average” models, respectively [41]. This ambiguity has been clarified in previous publications [42, 43]. Crucially, Lee & Nelder [43] highlighted that a conditional linear model can generate both a conditional and marginal mean effect [43]. Similarly, they conclude that the subsequent extrapolation from a marginal model average to a population average occurs as a result of the random cluster sampling, not of the chosen analytic model [43].

Overall, regardless of whether SW-CTs are analyzed with a mixed-effects or fixed-effects model, the corresponding model-specific estimators can be generally interpreted as the solutions to different estimating equations and can be compared. Furthermore, the estimators for both mixed-effects and fixed-effects models do not have an innately pre-determined inference space and both can be extended to a superpopulation framework, which typically yields simpler derivations with estimating equations or M-estimators [38], or a finite sample framework. To reiterate, it is the cluster random sampling itself that allows for the extrapolation of results from a given trial and its sampled clusters to the superpopulation of clusters [38], not the chosen analytic model.

### Simulation summary

Overall, we simulated 320 scenarios (5 SW-CT designs $\times$ 2 intervention effect sizes $\times$ 4 values of ${\tau }_{\alpha }^{2}$ $\times$ 2 cluster sizes $\times$ (1 no covariate imbalance or confounding condition + 1 covariate imbalance condition + 1 covariate confounding condition + 1 complete confounding condition)).

In each simulation scenario, we generated $s=\text{10,000}$ simulated data sets and estimated the intervention effect ${\widehat{\delta }}_{s}$ using each model described above. We present the properties of the intervention effect estimator in terms of bias, precision, power, coverage probability (CP), and root mean squared error (RMSE). We present the relative bias $(\text{Rel Bias}=[\text{Absolute bias}/\delta ]\times 100)$ when $\delta \ne 0$. Precision is the reciprocal of the average estimated variance $(\text{Precision}=1/\left[{\sum }_{s=1}^{\text{10,000}}{\text{Var}(\widehat{\delta }}_{s})/\text{10,000}\right])$. Power is the empirical power (if $\delta \ne 0$) or empirical type I error rate (if $\delta =0$) for rejecting the null hypothesis of $\delta =0$ at the two-sided significance level of 0.05, calculated as the probability that the asymptotic normal approximation derived 95% confidence interval excludes 0. Similarly, CP is the probability that the 95% confidence interval contains the true effect. RMSE is the square root of the average squared difference between the estimated effect ${\widehat{\delta }}_{s}$ and the true effect $\delta$ over the 10,000 simulated data sets for each scenario $\left(\text{RMSE}=\sqrt{{{\sum }_{s=1}^{\text{10,000}}{[\widehat{\delta }}_{s}-\delta ]}^{2}/\text{10,000}}\right)$. The Monte Carlo standard errors (standard deviation of the 10,000 estimated intervention effects ${\widehat{\delta }}_{s}$ for each scenario) are included in the supplementary material (Additional File 1.iii).

The above method for calculating the power can be described as an “uncorrected” approach to determining the power, where inferences are made with the normal distribution and no degrees of freedom computation is considered [27]. However, small sample corrections have been previously demonstrated to effectively reduce type I error in mixed-effects model analyses of P-CRTs [27]. Accordingly, we additionally consider the power of the “Satterthwaite” [44] degree of freedom approximation with inference using the t-distribution and the “Kenward–Roger” [45] correction that additionally adjusts the covariance matrix [27], in the simulation scenarios with no cluster-level covariate imbalance or confounding (Section "No cluster-level covariate imbalance or confounding"). We compare the uncorrected mixed-effects model (unadjusted and covariate-adjusted), mixed-effects model (unadjusted and covariate-adjusted) with the Satterthwaite correction, mixed-effects model (unadjusted and covariate-adjusted) with the Kenward–Roger correction, and fixed-effects model in SW-CT designs with 3, 4, or 5 clusters across 2000 simulated datasets for each scenario.

Additionally, we further generated 30,000 simulated datasets for the 3 cluster, 4 period SW-CT conditions (described in Section "Simulating time-invariant cluster-level chance covariate imbalance") in scenarios with no cluster-level covariate imbalance or confounding. We do so to evaluate the bias produced by the mixed-effects model, covariate-adjusted mixed-effects model, and fixed-effects model estimators, given three different quantiles of chance covariate imbalance severity (Section "Cluster-level chance covariate imbalance"). In expectation, this produces around 10,000 simulated datasets for each of the 3 quantiles.

## Results

In this section, we present the simulation results for scenarios with no cluster-level covariate imbalance or confounding (Section "No cluster-level covariate imbalance or confounding"), chance cluster-level covariate imbalance (Section "Cluster-level chance covariate imbalance"), severe cluster-level covariate imbalance (Section "Cluster-level covariate confounding"), and complete cluster-level confounding (Section "Complete cluster-level confounding"). In addition to the included figures, the results are presented as tables in the supplementary material (Additional File 2).

### No cluster-level covariate imbalance or confounding

**Fig. 2:**
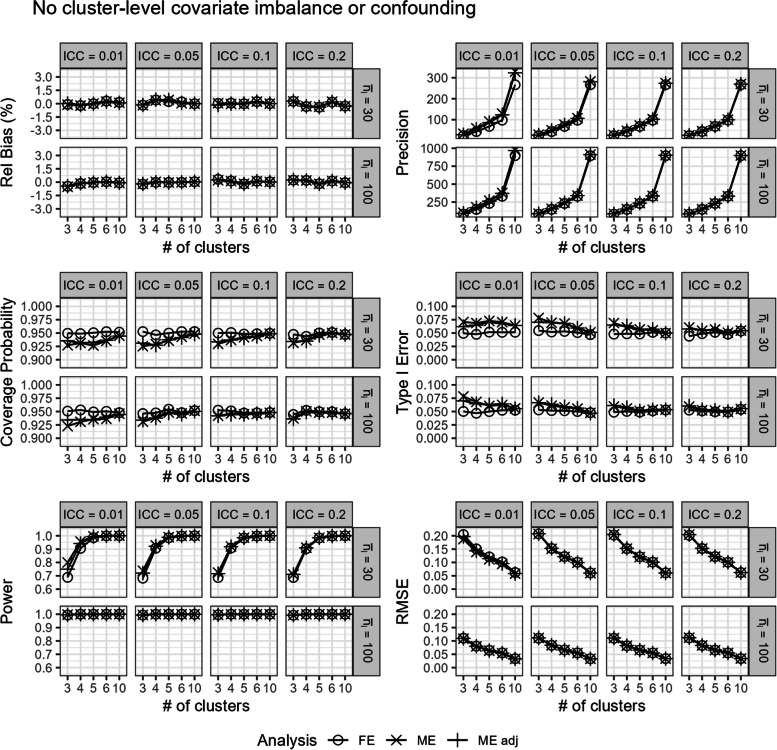
Comparison of the fixed-effects model (FE), mixed-effects model (ME), and covariate-adjusted mixed-effects model (ME adj) in terms of relative bias (%), precision, coverage probability, type I error, power, and root mean square error in scenarios with no cluster-level covariate imbalance or confounding. Results are presented across the number of clusters, ICC, and average cluster-period sample size

In scenarios with (i.) no cluster-level covariate imbalance or confounding, we observe that the fixed-effects and unadjusted and covariate-adjusted mixed-effects models were relatively unbiased (Fig. 2). Overall, the fixed-effects model had similar power and RMSE to the unadjusted and covariate-adjusted mixed-effects models (Fig. 2). The unadjusted and covariate-adjusted mixed-effects models often had higher precision and power than the fixed-effects model, especially when ICC was low (Fig. 2).

However, the unadjusted and covariate-adjusted mixed-effects models also had lower coverage probabilities and inflated type I errors with a small number of clusters or when the ICC was low (Fig. 2). This is especially true for the unadjusted mixed-effects model (Fig. 2). The coverage probabilities and type I error rates converge to the target level with the unadjusted and covariate-adjusted mixed-effects models when there were around six or more clusters (Fig. 2).

**Fig. 3:**
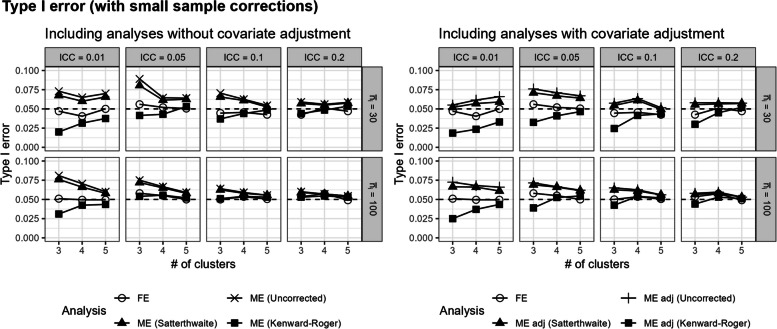
Type I error rates for the fixed-effects model, unadjusted and covariate-adjusted mixed-effects model (uncorrected), and unadjusted and covariate-adjusted mixed-effects model with a Satterthwaite or Kenward–Roger small sample size correction in scenarios with no cluster-level covariate imbalance or confounding. Results are presented from three to five clusters, ICC, and average cluster-period sample size. The expected type I error rate of 0.05 is shown with the dashed lines

In Fig. 3, the unadjusted and covariate-adjusted mixed-effects models with the Satterthwaite correction only slightly reduced the type I error rates compared to the uncorrected mixed-effects model, but still yielded inflated type I errors with a small number of clusters or when ICC was low. In contrast, the unadjusted and covariate-adjusted mixed-effects models with the Kenward–Roger correction could be overly conservative in analyses with small numbers of clusters and yield type I error rates that were too small (Fig. 3). In comparison, the fixed-effects model maintained the target level of coverage probability and type I error across all scenarios shown here (Figs. 2 and 3).

**Fig. 4:**
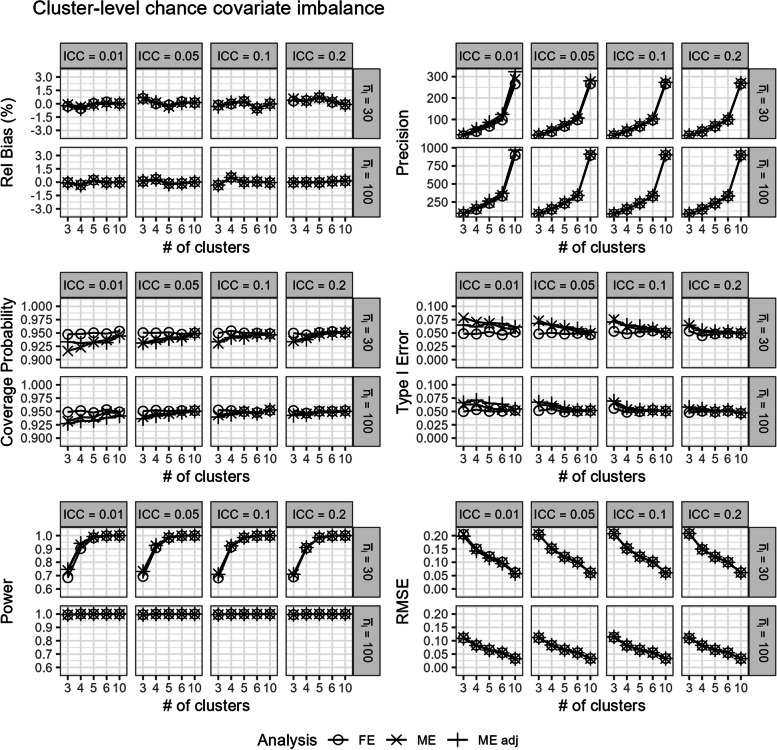
Alternative approaches for ICC estimation using a random effects model on the unexposed first period $j=1$ (RE (P1)), a mixed-effects model on the unexposed first period $j=1$ and always-exposed final period $j=J$ (ME (P1,PJ)), or a mixed-effects model on the full SW-CT data (ME (Full)). The average ICC estimates are presented with the true ICC values are shown with the dashed lines. The Monte Carlo standard errors of the ICC estimates over the simulation replicates are also presented. Results are presented across the number of clusters, ICC, and average cluster-period sample size

Overall, the three proposed approaches for estimating the ICC in a separate step from the fixed-effects model analyses (Section "Alternative approaches to ICC estimation in a SW-CT"), all mostly yield accurate estimates of the ICC with similar Monte Carlo standard errors for the ICC estimates across the simulation replicates (Fig. 4). Unsurprisingly, using more data to estimate the ICC, as seen with the full mixed-effects model (Eq. 2.1), yielded ICC estimates with lower Monte Carlo standard errors, and using the least data by analyzing only the first period from the SW-CT design with a random effects model yielded ICC estimates with the highest Monte Carlo standard errors (Fig. 4). With low ICC (equal to 0.01) and high ICC (equal to 0.2), the methods described could slightly overestimate or underestimate the true ICC, respectively (Fig. 4). This bias in ICC estimation has been previously described for mixed-effects models [46].

### Cluster-level chance covariate imbalance

**Fig. 5:**
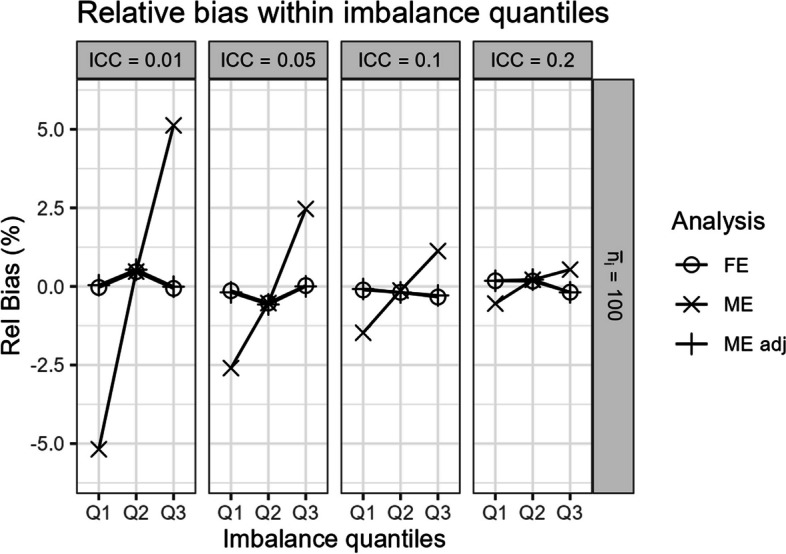
Comparison of the fixed-effects model (FE), mixed-effects model (ME), and covariate-adjusted mixed-effects model (ME adj) in terms of relative bias (%), precision, coverage probability, type I error, power, and root mean square error in scenarios with cluster-level chance covariate imbalance. Results are presented across the number of clusters, ICC, and average cluster-period sample size

**Fig. 6:**
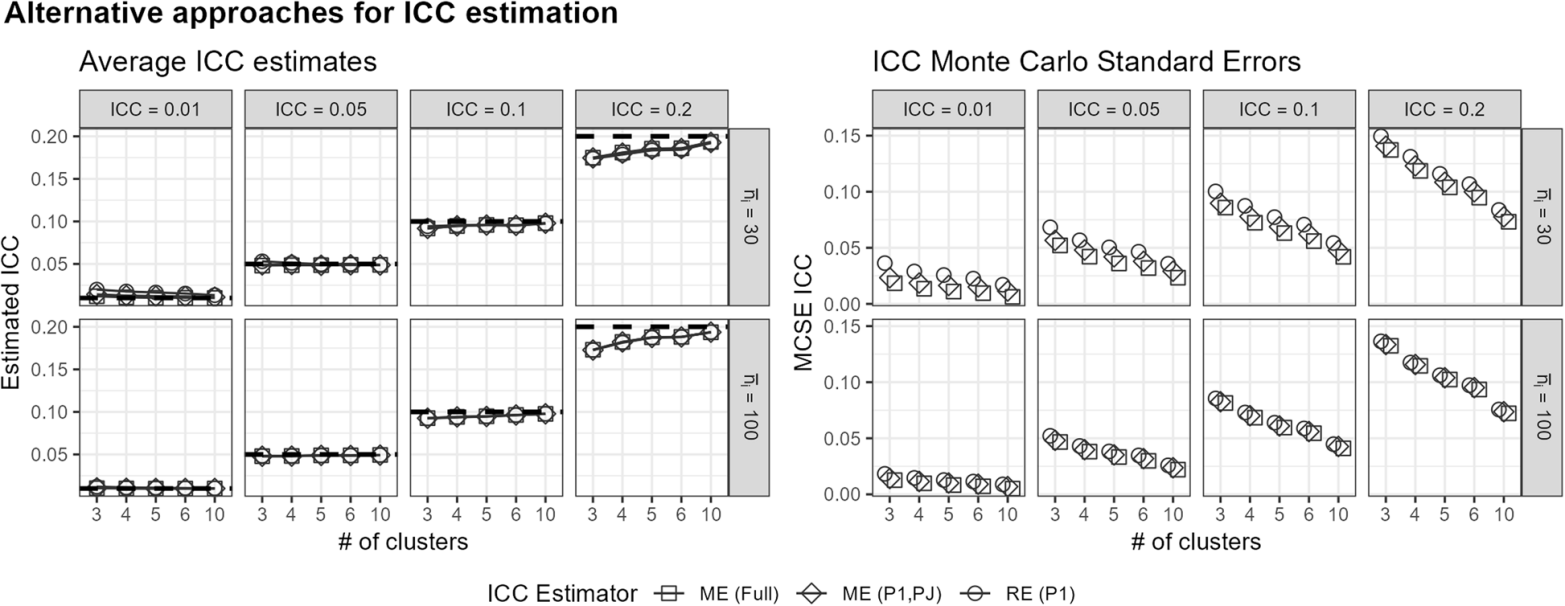
Relative bias from the fixed-effects model (FE), mixed-effects model (ME), and covariate-adjusted mixed-effects model (ME adj) within three quantiles of covariate imbalance severity (Q1: high negative imbalanced quantile, Q2: balanced quantile, Q3: high positive imbalanced quantile) in scenarios with three clusters and cluster-level chance covariate imbalance. Results are presented across different values of ICC with an average cluster-period sample size of 100

In scenarios with (ii.) chance cluster-level covariate imbalance, all models were generally unbiased (Fig. 5). Notably, Fig. 5 indicates that the unadjusted mixed-effects model is unbiased in expectation over the different quantiles of chance covariate imbalance severity. However, within the different potential quantiles of covariate imbalance, the unadjusted mixed-effects model can still be biased (Fig. 6). In Fig. 6, we graph the simulation results in a three-sequence, four-period SW-CT across scenarios as described in Section "Simulating time-invariant cluster-level chance covariate imbalance". Notably, the magnitude of bias within high negative (Q1) and positive (Q3) imbalanced quantiles are roughly equivalent in opposite directions (Fig. 6), hence yielding the unbiased mixed-effects model point estimates in expectation over the quantiles (Fig. 5). In contrast, the covariate-adjusted mixed-effects model and fixed-effects model both yield unbiased results in expectation over and within the different quantiles of covariate imbalance severity (Figs. 5 and 6).

Still, the mixed-effects model had largely similar performance in this scenario as compared to the previously discussed scenarios with no cluster-level covariate imbalance or confounding. However, we note a slight reduction in power in scenarios with covariate imbalance, low ICC, low number of clusters, and small cluster-period sample sizes (Figs. 2 and 5).

In addition to unbiased estimates within different quantiles of covariate imbalance, the covariate-adjusted mixed-effects model also has more precision and power than the unadjusted mixed-effects model and the fixed-effects model (Fig. 5). However, like the previous scenarios with no cluster-level covariate imbalance or confounding, the mixed-effects model with and without adjustment often had low coverage probabilities and inflated type I errors especially with a small number of clusters or when the ICC was low (Fig. 5). In contrast, the fixed-effects model continued to maintain good coverage probability and correct type I error control (Fig. 5). Overall, the fixed-effects model had similar power and RMSE to the mixed-effects model with and without adjustment (Fig. 5).

### Cluster-level covariate confounding

**Fig. 7:**
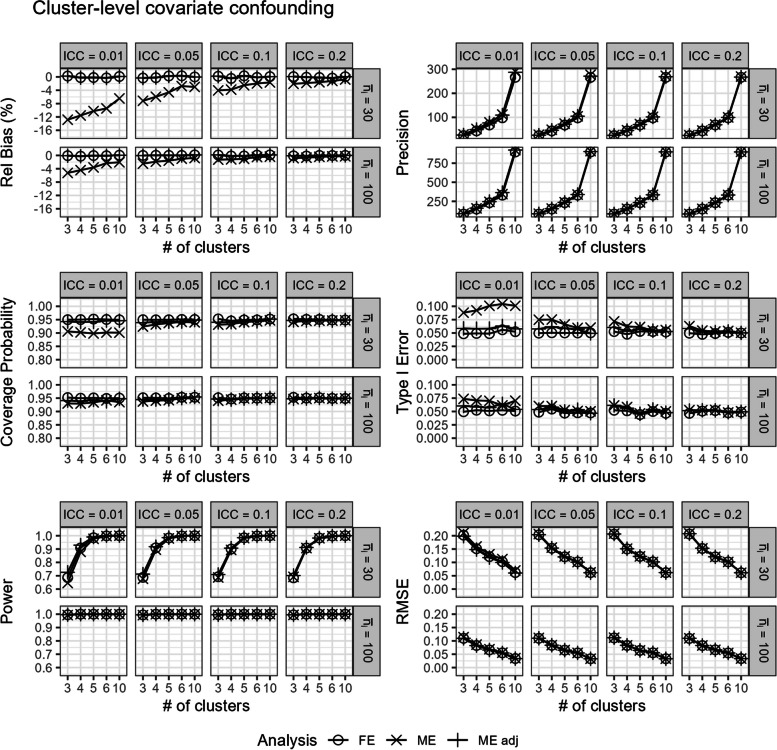
Comparison of the fixed-effects model (FE), mixed-effects model (ME), and covariate-adjusted mixed-effects model (ME adj) in terms of relative bias (%), precision, coverage probability, type I error, power, and root mean square error in scenarios with cluster-level covariate confounding. Results are presented across number of clusters, ICC, and average cluster-period sample size

In scenarios with (iii.) cluster-level covariate confounding, the mixed-effects model without adjustment produced biased estimates especially when the ICC was small (Fig. 7). Given the cluster-level covariate confounding, both the validity and power of the unadjusted mixed-effects model is impacted. As a result of this bias, the mixed-effects model can yield results with very poor coverage probability, type I error rates, and power (Fig. 7). Meanwhile, both the covariate-adjusted mixed-effects model and the fixed-effects model produced unbiased estimates (Fig. 7).

Notably, the covariate-adjusted mixed-effects model can still have low coverage probabilities and inflated type I errors, especially with a small number of clusters or when the ICC was low (Fig. 7). In contrast, the fixed-effects model maintained relatively unbiased estimates with good coverage probability and correct type I error control (Fig. 7). Overall, the fixed-effects model also had similar power and RMSE to the mixed-effects model with adjustment (Fig. 7).

### Complete cluster-level confounding

**Fig. 8:**
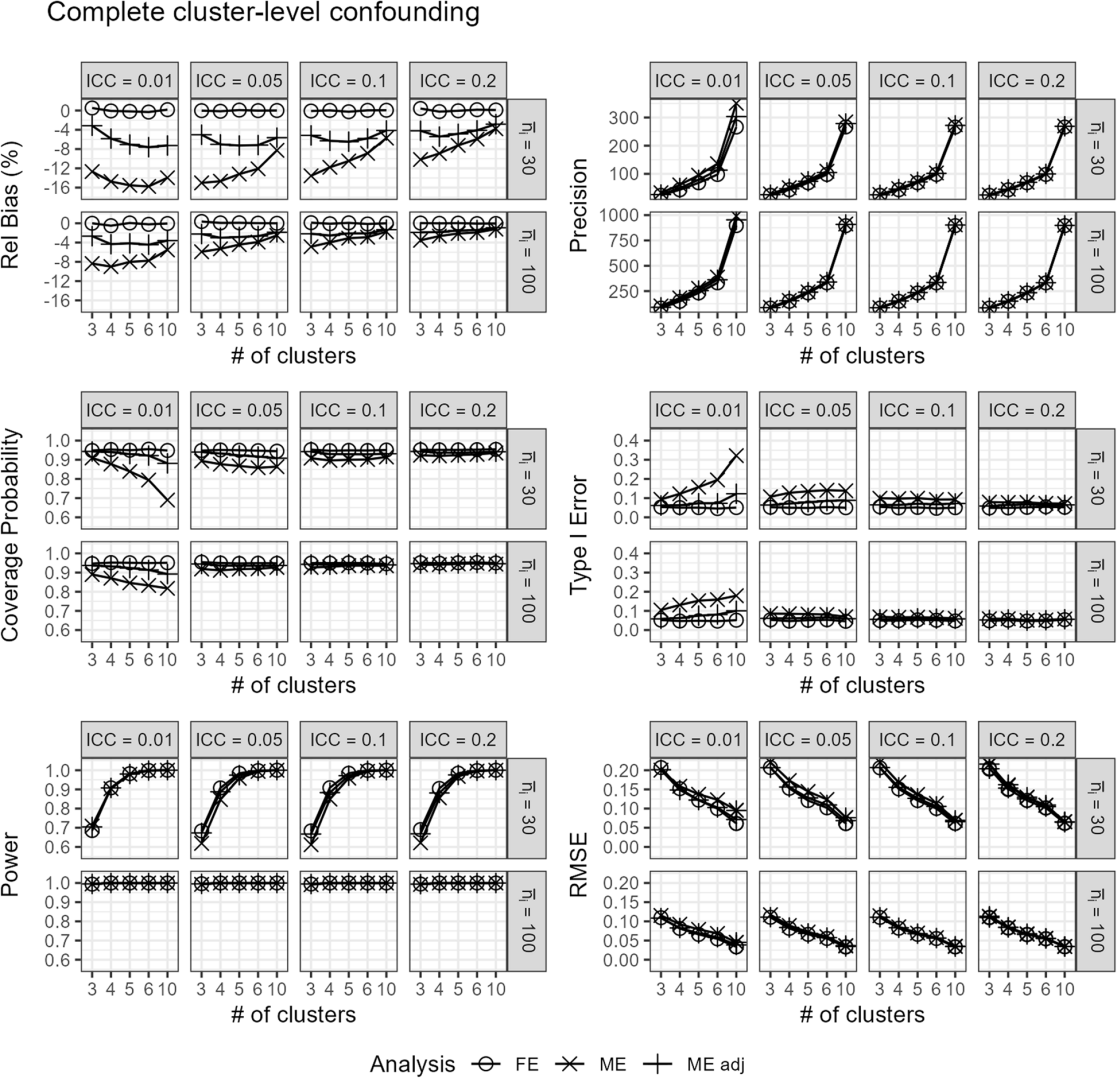
Comparison of the fixed-effects model (FE), mixed-effects model (ME), and covariate-adjusted mixed-effects model (ME adj) in terms of relative bias (%), precision, coverage probability, type I error, power, and root mean square error in scenarios with complete cluster-level confounding. Results are presented across number of clusters, ICC, and average cluster-period sample size

In scenarios with (iv.) complete cluster-level confounding, the unadjusted mixed-effects model produced very biased estimates, especially when the number of clusters or ICC was small (Fig. 8). The covariate-adjusted mixed-effects model, adjusting for the confounding covariate as an imperfect proxy indicator for the complete cluster-level confounding (Section "Simulating complete cluster-level confounding"), abated some of this bias but notably still yielded very biased results. This large bias in the mixed-effects model estimates led to poor coverage probabilities and very inflated type I error rates (Fig. 8). In contrast, the fixed-effects model produced unbiased estimates with good coverage probabilities and correct type I error rates (Fig. 8). Overall, the fixed-effects model also had similar power and lower RMSE compared to the mixed-effects models (Fig. 8).

In these scenarios with complete cluster-level confounding, the three proposed approaches for estimating the ICC in a separate step from the analyses all mostly yielded accurate estimates of the ICC (Additional File 1.ii), with results corresponding to those observed in Fig. 4. Despite the treatment effect estimates being biased by the improper use of a mixed-effects model in such a scenario, we observe that the mixed-effects model can still yield nearly unbiased estimates of the ICC.

## Discussion

This article has two primary goals, first to establish that the fixed-effects model can yield results with proper type I error rates, in contrast to mixed-effects models, when the number of clusters is low in SW-CTs with continuous outcomes. Second, we explicitly demonstrate that the fixed-effects method automatically adjusts for cluster-level covariate imbalance and confounding. Additionally, we address some misconceptions regarding the inferential target for the mixed-effects and fixed-effects models.

We show that the mixed-effects model can be vulnerable to low coverage probabilities and inflated type I error rates in cross-sectional SW-CTs with continuous outcomes, especially when there are fewer than six clusters or when the ICC is low. Such overly narrow confidence intervals have previously been demonstrated in P-CRTs with a small number of clusters [26, 27] and have now been similarly demonstrated in SW-CTs. Furthermore, the mixed-effects models with Satterthwaite or Kenward–Roger small sample corrections can lead to inflated or overly conservative type I errors, respectively. In contrast, the fixed-effects model consistently produced good coverage probability and type I error rates without any meaningful compromises to power across all SW-CT scenarios explored in this article. These small sample correction results were primarily demonstrated in simulation scenarios with (i.) no cluster-level covariate imbalance or confounding. Since these corrections primarily include a degree of freedom adjustment and maybe a mixed-effects model variance estimator adjustment (with Kenward–Roger), these observed results are expected to extend to other simulation scenarios where the unadjusted or covariate-adjusted mixed-effects models are expected to yield unbiased results.

Previous ambiguity over the application of covariate adjustment methods has been attributed to a “misunderstanding on the part of trialists that clusters serve as their own controls and that imbalances are therefore a lesser concern” [5]. However, this is primarily a misunderstanding in the context of mixed-effects model analyses, which conceptually make within-period comparisons between-clusters while adjusting for the intracluster correlation [47]. In contrast, the fixed-effects model does actually make within-cluster comparisons, with each cluster serving as their own control [24].

When there is cluster-level chance covariate imbalance, the mixed-effects model without adjustment is unbiased in expectation over the different potential levels of imbalance. However, we clarify that the mixed-effects model can still be conditionally biased within different quantiles of covariate imbalance severity. In contrast, the covariate-adjusted mixed-effects model and fixed-effects model both yield unbiased results in expectation over and within the different quantiles of covariate imbalance. An analogous result was previously reported in P-CRTs with cluster-level chance covariate imbalance [3]. In this article, we demonstrate this result with a three-cluster, four-period SW-CT example which allows us to simply define the three different quantiles of covariate imbalance severity. It is notably more complicated to define the different quantiles of covariate imbalance severity in larger SW-CT designs; however, the results are expected to extend to larger designs.

When there is cluster-level covariate imbalance or confounding, the covariate-adjusted mixed-effects model produces minimally biased estimates. However, this requires that the imbalanced covariate be known and measured, which may not always be practical. Furthermore, in scenarios with complete cluster-level confounding, the mixed-effects model yields very biased estimates. Even a covariate-adjusted mixed-effects model, where the adjusted covariate serves as an imperfect proxy indicator for the covariate imbalance, yielded very biased estimates. In contrast, the fixed-effects model automatically accounts for all measured and unmeasured time-invariant cluster-level imbalanced covariates and confounding to robustly yield unbiased estimates.

Additionally, we also observed that the basic Hausman test has incredibly low power in SW-CTs, despite the presence of complete cluster-level confounding (Additional File 1.iv). We do not advise the use of the Hausman test to judge whether there is cluster-level confounding in SW-CTs. Future work should explore alternative tests that may be more powerful in detecting such cluster-level confounding.

Such cluster-level confounding in SW-CTs, although rare given randomization, may still occur. For example, Groshaus et al. [15] reported on a trial where practical and scheduling issues prevented randomization [15]. DiDiodato et al. (2015) reported on a trial where randomization did not occur due to ethical and logistical reasons [48]. In practice, non-randomized SW-CTs have also been implemented to evaluate the effects of a mammography intervention [16], evidence-based quality improvement interventions [17], and a prospective frontline surveillance system [18]. Furthermore, unmeasured cluster-level confounding can still occur in SW-CRTs in the form of post-randomization selection biases, especially as subjects are often recruited after cluster randomization has already occurred [49, 50]. However, there are further potential issues that may arise from post-randomization selection bias in CRTs [49, 50], with such topics being beyond the scope of this current work.

### Limitations

Previous work in P-CRTs [51] and SW-CTs [4] has also explored individual-level imbalanced covariates alongside cluster-level imbalanced covariates. SW-CTs with small numbers of clusters are also at risk of individual-level covariate imbalance [5]. However, we do not explore methods for dealing with individual-level covariate imbalance. In this article, we only simulated cluster-level covariate imbalance to focus on how specifying cluster intercepts as fixed in a fixed-effects model can automatically adjust for these imbalances in a SW-CT.

Furthermore, we only simulated SW-CT designs where all clusters receive both the control and intervention by the end of the trial. Some alternative SW-CT designs have been proposed, including the “optimized design” which includes unexposed and always-exposed clusters to optimize the efficiency of the SW-CT under analysis with the mixed-effects model (Eq. 2.1) [52, 53]. Previous work has demonstrated that such designs can still be analyzed with a fixed-effects model (Eq. 3), where unexposed and always-exposed clusters will still help improve the efficiency of the intervention effect estimator [20]. We do not include or explore the implications of covariate imbalance in SW-CTs with such “optimized designs” in this article.

A natural mixed-effects model extension that has become increasingly popular in practice is the “Hooper-Girling” mixed-effects model, which includes an additional cluster-period random interaction term to induce a nested-exchangeable correlation structure [52, 54]. We do not evaluate such nested data-generating processes or analysis models in this article. The fixed-effects model can be easily extended to have a similar nested correlation structure between periods within clusters by including a cluster-period random interaction term alongside the cluster fixed effect. The properties of such a nested fixed-effects model have not yet been explicitly evaluated in the context of SW-CTs but have been proposed in the analysis of cluster randomized cross-over trials (CRXO) [55].

The CONSORT extension to SW-CRTs suggests including the ICC estimates when reporting results to help inform future studies [35]. Unlike the mixed-effects model, the fixed-effects model does not automatically estimate the between-cluster variance and ICC [34]. However, there are alternative ways to estimate the ICC while using a fixed-effects model. In this article, we proposed estimating the ICC in a separate step from the analysis of the treatment effect, by using a random-effects or mixed-effects model on different subsets of the SW-CT data. Furthermore, we can easily extend this ICC estimation step to estimate the between-period and within-period ICC’s by including a cluster-period random interaction term in the described mixed-effects models. Notably, the ICC estimation approaches using mixed-effects models implicitly assume a constant treatment effect within the final period, which may not always be the case in SW-CTs [56, 57]. Still, even in scenarios with complete cluster-level confounding, the three proposed approaches for estimating the ICC in a separate step from the analyses all mostly yielded accurate estimates of the ICC (Additional File 1.ii), with results corresponding to those observed in Fig. 4. Despite the treatment effect estimates being biased by the improper use of a mixed-effects model in such a scenario, we observe that the mixed-effects model can still yield nearly unbiased estimates of the ICC. Future research may further explore and validate alternative methods for calculating the ICC in fixed-effects models for SW-CTs.

Previous work has discussed the “design effect” (the variance inflation factor) that describes the amount by which the cluster randomized trial sample size needs to increase relative to a similarly designed individually randomized trial [58]. This design effect, as previously described [58], was derived from the Hussey & Hughes mixed-effects model (Eq. 2.1) and simply characterizes the amount of variance inflation resulting from specifying an exchangeable correlation structure (in a cluster randomized trial) as opposed to an independence correlation structure (in an individually randomized trial). Here, we discuss the strengths of the fixed-effects model as an alternative to the mixed-effects model for the analysis of SW-CTs. While we do not derive the design effect for the fixed-effects model in this article, our results indicate that the fixed-effects model has a marginally higher variance (and lower precision) than the mixed-effects model, implying that the fixed-effects model would have a similar but slightly higher design effect than that which was previously described for the mixed-effects model. Future work can look to derive a similar design effect in the fixed-effects model described here and in the fixed-effects model with an additional cluster-period random interaction term and induced nested correlation structure.

In this article, we have described how the fixed-effects model controls for all cluster-level time-invariant variables. We outline the benefits of this in terms of controlling for unmeasured imbalanced covariates and confounding variables. However, such cluster-level time-invariant covariates may sometimes be of interest (e.g., distance of a village from the nearest health center) and cannot be explicitly modelled in such fixed-effects models [24]. Additionally, some recent publications have illuminated potential shortcomings of the fixed-effects model with heterogeneous treatment effects [59–61]. Notably, these shortcomings are not unique to the fixed-effects model. Indeed, the standard mixed-effects model with an immediate and constant treatment effect can have similar issues when the treatment effect structure is misspecified [47, 56].

A notable extension to the mixed-effects model replaces the constant intervention effect with time-on-intervention effects, where separate discrete intervention effects are specified for each elapsed period of exposure time since the intervention was first introduced to a cluster [56, 57, 62, 63]. Ma et al. recently reported that the benefits of pre-balancing with a measured imbalanced cluster-level covariate were particularly heightened when such time-on-intervention effects (which they referred to as a “learning effect”) were present in the SW-CT [13]. In this article, we simulate data assuming a constant and immediate intervention effect and do not consider such time-on-intervention effects. Future work can explore the properties of the fixed-effects model with specified time-on-intervention effects.

In this work, the mixed-effects and fixed-effects models are described for individual-level responses rather than cluster-level responses. However, the models described here can be easily extended to target a cluster-level response by implementing inverse cluster size or cluster-period size weighting [30, 31, 42]. Alternatively, analyses may also be applied on the cluster period means to target the cluster-level response [6, 27]. Regardless, in the presence of non-informative cluster sizes, the individual and cluster-average treatment effect estimands are expected to coincide, and unweighted and weighted mixed-effects and fixed-effects models are anticipated to be unbiased and consistent for these estimands [42]. Otherwise, in the presence of informative cluster sizes, the properties of fixed-effects and mixed-effects models have been observed to differ when targeting the individual and cluster-average treatment effect estimands in other multi-period cluster trial designs [31], adding another level of consideration when comparing these two models. The properties of these models in the presence of informative cluster sizes have not yet been clearly evaluated in SW-CT designs and can be the target of future work.

## Conclusion

In this article, we fill gaps in the SW-CT literature concerning the performance of the fixed-effects model in maintaining correct type I error and automatically adjusting for all cluster-level covariate imbalance and confounding. We demonstrate that the mixed-effects model can be vulnerable to poor coverage probabilities and inflated type I error rates in the analyses of cross-sectional SW-CTs with continuous outcomes and a small number of clusters. Furthermore, the mixed-effects model requires imbalanced covariates to be measured. This may not always be practical. In contrast, the fixed-effects model is a more robust analytic model for SW-CTs with a small number of clusters and if cluster-level time-invariant covariate imbalance or confounding is suspected.

## Supplementary Information

Additional file 1. Additional Simulation Results: (i.) Conditional independence of observations within cluster in fixed effects and mixed effects models, (ii.) Alternative approaches for ICC estimation in scenarios with complete cluster-level confounding, (iii.) Monte Carlo standard errors presented across simulation scenarios, number of clusters, ICC, and average cluster-period sample size, (iv.) Power of the Hausman test for detecting cluster-level confounding in scenarios where such confounding exists.

Additional file 2. Table of Simulation Results: Results (aside from Type I error) are presented for scenarios where $\delta=0.5$.

Additional file 3. R code for simulation, analysis, and plotting.

## Data Availability

The corresponding R code is available in Additional file 3.

## Abbreviations

CRT: Cluster randomized trial
P-CRT: Parallel cluster randomized trial
SW-CRT: Stepped-wedge cluster randomized trial
SW-CT: Stepped-wedge cluster trial
FE: Fixed effects
ME: Mixed effects
ICC: Intra-cluster correlation coefficient
RMSE: Root mean squared error
CP: Coverage probability
