# Supplementary material for: The fixed-effects model for robust analysis of stepped-wedge cluster trials with a small number of clusters and continuous outcomes: a simulation study
Source: Trials. 2024 Oct 25;25:718. doi: 10.1186/s13063-024-08572-1 (PMC11515801; doi:10.1186/s13063-024-08572-1)
Supplement: Supplementary file 1 — Additional file 1. Additional Simulation Results: (i.) Conditional independence of observations within cluster in fixed effects and mixed effects models, (ii.) Alternative approaches for ICC estimation in scenarios with complete cluster-level confounding, (iii.) Monte Carlo standard errors presented across simulation scenarios, number of clusters, ICC, and average cluster-period sample size, (iv.) Power of the Hausman test for detecting cluster-level confounding in scenarios where such confounding exists. [file 13063_2024_8572_MOESM1_ESM.docx]

# Additional File 1

(i.) Conditional independence of observations within cluster in fixed effects and mixed effects models.

(i.a.) In a fixed effects model where:

$$Y_{ijk}=\phi_{j}+X_{ij}\delta+\alpha_{i}+e_{ijk}$$

$$e_{ijk}\overset{iid}{\sim}N(0,\sigma_{w}^{2})$$

as described in Equation 3 in Section 2.3.2 of the main article text.

Given two observations $\left( Y_{ips},Y_{iqr} \right)$ from within the same cluster $i$, where $p,q\in\left[ 1,\ldots,J \right]$ and $s\in\left[ 1,\ldots,K_{ip} \right],r\in\left[ 1,\ldots,K_{iq} \right]$. If $p=q,s\neq r$ (not the same individual). Accordingly, the conditional covariance of two observations $\left( Y_{ips},Y_{iqr} \right)$ from within the same cluster (eg. $\alpha_{1}$) observed is:

$$Cov\left( Y_{ips},Y_{iqr} | \alpha_{1} \right)=E\left[ Y_{ips},Y_{iqr}|\alpha_{1} \right]-E\left[ Y_{ips}|\alpha_{1} \right]E\left[ Y_{iqr}|\alpha_{1} \right]$$

$$=E\left[ \left( \phi_{p}+X_{ip}\delta+\alpha_{i}+e_{ips} \right)\left( \phi_{q}+X_{iq}\delta+\alpha_{i}+e_{iqr} \right)|\alpha_{1} \right]-E\left[ \phi_{p}+X_{ip}\delta+\alpha_{i}+e_{ips}|\alpha_{1} \right]E\left[ \phi_{q}+X_{iq}\delta+\alpha_{i}+e_{iqr}|\alpha_{1} \right]$$

$$=E\left[ \left( \phi_{p}+X_{1p}\delta+\alpha_{1}+e_{1ps} \right)\left( \phi_{q}+X_{1q}\delta+\alpha_{1}+e_{1qr} \right) \right]-E\left[ \phi_{p}+X_{1p}\delta+\alpha_{1}+e_{1ps} \right]E\left[ \phi_{q}+X_{1q}\delta+\alpha_{1}+e_{1qr} \right]$$

$$=E\left[ \left( \phi_{p}+X_{1p}\delta+\alpha_{1}+e_{1ps} \right)\left( \phi_{q}+X_{1q}\delta+\alpha_{1}+e_{1qr} \right) \right]-E\left[ \phi_{p}+X_{1p}\delta+\alpha_{1}+e_{1ps} \right]E\left[ \phi_{q}+X_{1q}\delta+\alpha_{1}+e_{1qr} \right]$$

$$=\left( \phi_{p}+X_{1p}\delta+\alpha_{1} \right)\left( \phi_{q}+X_{1q}\delta+\alpha_{1} \right)+\left( \phi_{p}+X_{1p}\delta+\alpha_{1} \right)E\left[ e_{1qr} \right]+E\left[ e_{1ps} \right]\left( \phi_{q}+X_{1q}\delta+\alpha_{1} \right)+E\left[ e_{1ps}e_{1qr} \right]-\left( \phi_{p}+X_{1p}\delta+\alpha_{1} \right)\left( \phi_{q}+X_{1q}\delta+\alpha_{1} \right)-\left( \phi_{p}+X_{1p}\delta+\alpha_{1} \right)E\left[ e_{1qr} \right]-E\left[ e_{1ps} \right]\left( \phi_{q}+X_{1q}\delta+\alpha_{1} \right)-E\left[ e_{1ps} \right]E\left[ e_{1qr} \right]$$

$$=E\left[ e_{1ps}e_{1qr} \right]- E\left[ e_{1ps} \right]E\left[ e_{1qr} \right]$$

$$=E\left[ e_{1ps} \right]E\left[ e_{1qr} \right]- E\left[ e_{1ps} \right]E\left[ e_{1qr} \right]$$

$$=0$$

with standard assumptions of the described fixed effects model (Equation 3). Therefore, observations within a cluster in a fixed effects model, given a specific cluster fixed intercept, are conditionally independent.

(i.b.) In a mixed effects model where:

$$Y_{ijk}=\phi_{j}+X_{ij}\delta+\alpha_{i}+e_{ijk}$$

$$\alpha_{i}\overset{iid}{\sim}N\left( 0, \tau_{\alpha}^{2} \right)$$

$$e_{ijk}\overset{iid}{\sim}N\left( 0,\sigma_{w}^{2} \right).$$

as described in Equation 2.1 in Section 2.3.1 of the article.

Again, given two observations $\left( Y_{ips},Y_{iqr} \right)$ from within the same cluster $i$, where $p,q\in\left[ 1,\ldots,J \right]$ and $s\in\left[ 1,\ldots,K_{ip} \right],r\in\left[ 1,\ldots,K_{iq} \right]$. If $p=q,s\neq r$ (not the same individual). Accordingly, the unconditional covariance of two observations $\left( Y_{ips},Y_{iqr} \right)$ from within the same cluster (eg. $\alpha_{1}$) observed is:

$$Cov\left( Y_{ips},Y_{iqr} \right)=E\left[ Y_{ips},Y_{iqr} \right]-E\left[ Y_{ips} \right]E\left[ Y_{iqr} \right]$$

$$=E\left[ \left( \phi_{p}+X_{ip}\delta+\alpha_{i}+e_{ips} \right)\left( \phi_{q}+X_{iq}\delta+\alpha_{i}+e_{iqr} \right) \right]-E\left[ \phi_{p}+X_{ip}\delta+\alpha_{i}+e_{ips} \right]E\left[ \phi_{q}+X_{iq}\delta+\alpha_{i}+e_{iqr} \right]$$

$$=\left( \phi_{p}+X_{1p}\delta\right)\left( \phi_{q}+X_{1q}\delta\right)+\left( \phi_{p}+X_{1p}\delta\right)E\left[ \alpha_{i} \right]+E\left[ \alpha_{i} \right]\left( \phi_{q}+X_{1q}\delta\right)+E\left[ \alpha_{i}^{2} \right]+\left( \phi_{p}+X_{1p}\delta\right)E\left[ e_{1qr} \right]+E\left[ e_{1ps} \right]\left( \phi_{q}+X_{1q}\delta\right)+E\left[ e_{1ps}e_{1qr} \right]-\left( \phi_{p}+X_{1p}\delta\right)\left( \phi_{q}+X_{1q}\delta\right)-\left( \phi_{p}+X_{1p}\delta\right)E\left[ \alpha_{i} \right]-E\left[ \alpha_{i} \right]\left( \phi_{q}+X_{1q}\delta\right)-E\left[ \alpha_{i} \right]E\left[ \alpha_{i} \right]-\left( \phi_{p}+X_{1p}\delta\right)E\left[ e_{1qr} \right]-E\left[ e_{1ps} \right]\left( \phi_{q}+X_{1q}\delta\right)-E\left[ e_{1ps} \right]E\left[ e_{1qr} \right]$$

$$=E\left[ \alpha_{i}^{2} \right]-E\left[ \alpha_{i} \right]E\left[ \alpha_{i} \right]=Var\left( \alpha_{i} \right)=\tau_{\alpha}^{2}$$

with standard assumptions of the described mixed effects model (Equation 2.1). Therefore, observations within a cluster in a mixed effects model are correlated.

However, the conditional covariance of two observations $\left( Y_{ips},Y_{iqr} \right)$ from within the same cluster (eg. $\alpha_{1}$) is:

$$Cov\left( Y_{ips},Y_{iqr} | \alpha_{1} \right)=E\left[ Y_{ips},Y_{iqr}|\alpha_{1} \right]-E\left[ Y_{ips}|\alpha_{1} \right]E\left[ Y_{iqr}|\alpha_{1} \right]$$

$$=E\left[ \left( \phi_{p}+X_{ip}\delta+\alpha_{i}+e_{ips} \right)\left( \phi_{q}+X_{iq}\delta+\alpha_{i}+e_{iqr} \right)|\alpha_{1} \right]-E\left[ \phi_{p}+X_{ip}\delta+\alpha_{i}+e_{ips}|\alpha_{1} \right]E\left[ \phi_{q}+X_{iq}\delta+\alpha_{i}+e_{iqr}|\alpha_{1} \right]$$

$$=E\left[ \left( \phi_{p}+X_{1p}\delta+\alpha_{1}+e_{1ps} \right)\left( \phi_{q}+X_{1q}\delta+\alpha_{1}+e_{1qr} \right) \right]-E\left[ \phi_{p}+X_{1p}\delta+\alpha_{1}+e_{1ps} \right]E\left[ \phi_{q}+X_{1q}\delta+\alpha_{1}+e_{1qr} \right]$$

$$=E\left[ \left( \phi_{p}+X_{1p}\delta+\alpha_{1}+e_{1ps} \right)\left( \phi_{q}+X_{1q}\delta+\alpha_{1}+e_{1qr} \right) \right]-E\left[ \phi_{p}+X_{1p}\delta+\alpha_{1}+e_{1ps} \right]E\left[ \phi_{q}+X_{1q}\delta+\alpha_{1}+e_{1qr} \right]$$

$$=\left( \phi_{p}+X_{1p}\delta+\alpha_{1} \right)\left( \phi_{q}+X_{1q}\delta+\alpha_{1} \right)+\left( \phi_{p}+X_{1p}\delta+\alpha_{1} \right)E\left[ e_{1qr} \right]+E\left[ e_{1ps} \right]\left( \phi_{q}+X_{1q}\delta+\alpha_{1} \right)+E\left[ e_{1ps}e_{1qr} \right]-\left( \phi_{p}+X_{1p}\delta+\alpha_{1} \right)\left( \phi_{q}+X_{1q}\delta+\alpha_{1} \right)-\left( \phi_{p}+X_{1p}\delta+\alpha_{1} \right)E\left[ e_{1qr} \right]-E\left[ e_{1ps} \right]\left( \phi_{q}+X_{1q}\delta+\alpha_{1} \right)-E\left[ e_{1ps} \right]E\left[ e_{1qr} \right]$$

$$=E\left[ e_{1ps}e_{1qr} \right]- E\left[ e_{1ps} \right]E\left[ e_{1qr} \right]$$

$$=E\left[ e_{1ps} \right]E\left[ e_{1qr} \right]- E\left[ e_{1ps} \right]E\left[ e_{1qr} \right]$$

$$=0$$

with standard assumptions of the described mixed effects model (Equation 2.1). Therefore, observations within a cluster in the described mixed effects model, given a specific cluster random intercept, are conditionally independent.

(ii.) Alternative approaches for ICC estimation in scenarios with complete cluster-level confounding using a random effects model on the unexposed first period $j=1$ (RE (P1)), a mixed effects model on the unexposed first period $j=1$ and always-exposed final period $j=J$ (ME (P1,PJ)), or a mixed effects model on the full SW-CT data (ME (Full)). The average ICC estimates are presented with the true ICC values are shown with the dashed lines. The Monte Carlo standard errors of the ICC estimates over the simulation replicates are also presented. Results are presented across number of clusters, ICC, and average cluster-period sample size.


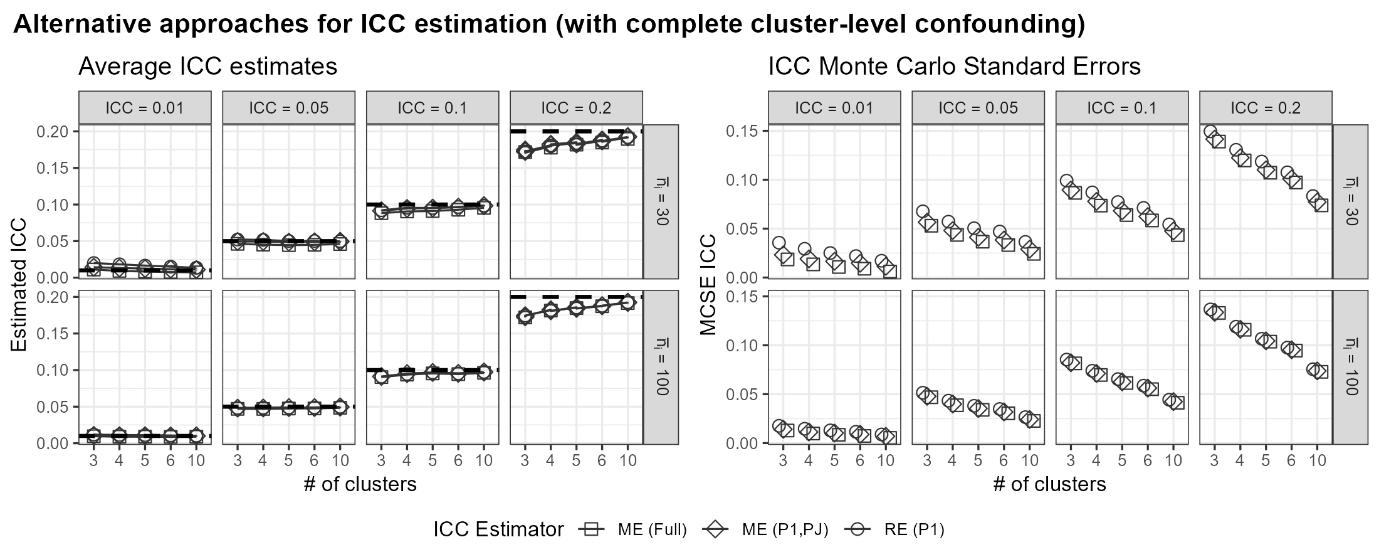


(iii.) Monte Carlo standard errors presented across simulation scenarios, number of clusters, ICC, and average cluster sample size.


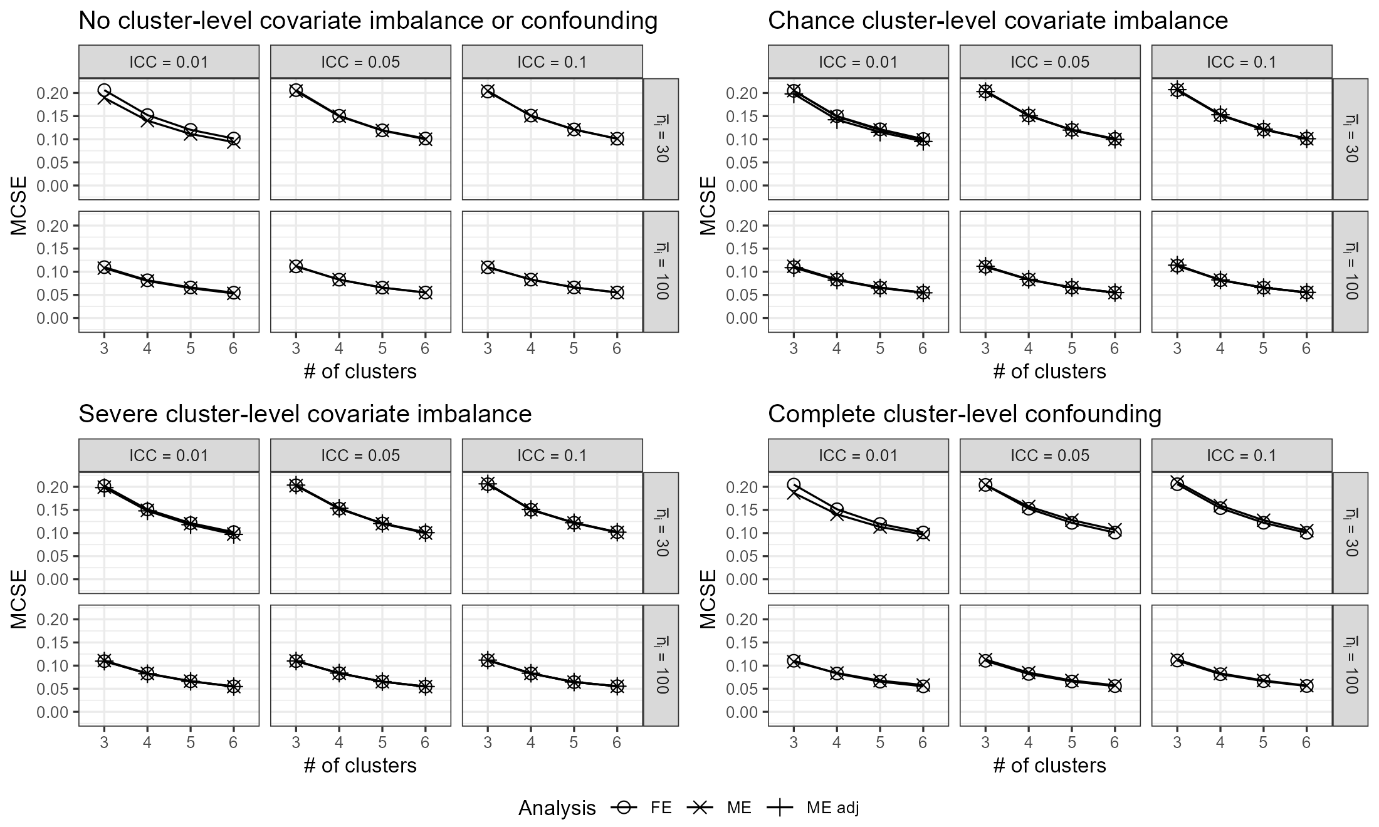


(iv.) Hausman Test

A major difference between modeling cluster intercepts as random or fixed depends on whether the cluster intercepts are correlated with the other model covariates (Gardiner et al., 2009). Mixed effects models treat clusters as random intercepts that are uncorrelated with both the residual error term and other model covariates (commonly referred to as “exogeneity”) (Hausman, 1978). If the cluster random intercepts are correlated with the other model covariates (commonly referred to as “endogeneity”), as is the case when there are unmeasured cluster-level time-invariant confounders, the mixed effects intervention effect estimator becomes biased, inconsistent, and fails to remove the confounding (Gardiner et al., 2009; Hausman, 1978; Wooldridge, 2010).

We can rewrite both the mixed effects model (Equations 2.1) and fixed effects model (Equation 3) as:

$$Y_{ijk}={\phi_{j}+X}_{ij}\delta+\alpha_{i}+e_{ijk}$$

$$=W_{ijk}\Theta+\alpha_{i}+e_{ijk}$$

where $\phi_{j}$ are the $J$ fixed effects for each period and $\alpha_{i}$ are the random or $I-1$ fixed cluster deviations. Then $W_{ijk}$ is a $(\sum_{i=1}^{I} \sum_{j=1}^{J} n_{ij})$ by $\left( J+1 \right)$ design matrix and $\Theta$ is a $(J+1)$ by 1 vector of the parameters, $(\phi_{1},\ldots,\phi_{J},\delta)'$.

When $W_{ijk}$ is correlated with $\alpha_{i}$, as is the case when there is cluster-level confounding, the mixed effects model estimators are no longer consistent. The Hausman test (Hausman, 1978) was proposed to detect such endogeneity and confounding by testing the differences between the model coefficients $\hat{\Theta}_{ME}$ as estimated by a mixed effects model (Equation 2.1) against the corresponding coefficients $\hat{\Theta}_{FE}$ in a fixed effects model (Equation 3). Generally, the Hausman test statistic is:

$$H=\left( \hat{\Theta}_{FE}-\hat{\Theta}_{ME} \right)^{'}\left[ VarCov\left( \hat{\Theta}_{FE} \right)-VarCov\left( \hat{\Theta}_{ME} \right) \right]^{-1}\left( \hat{\Theta}_{FE}-\hat{\Theta}_{ME} \right)$$

following a $\chi_{df}^{2}$ distribution where $df=rank\left[ Var\left( \hat{\Theta}_{FE} \right)-Var\left( \hat{\Theta}_{ME} \right) \right]=J+1$. In Section 3.4, we will present the power of the Hausman test for simulation scenarios with complete cluster-level confounding.

We explored the power of the Hausman test for detecting cluster-level confounding in scenarios where such confounding exists. In the scenarios with complete cluster-level confounding, the Hausman test had almost no power for detecting the confounding, despite the biased mixed effects model intervention effect estimates (Main Manuscript Figure 8). The Hausman test is generally unable to detect when there is cluster-level confounding in SW-CTs.


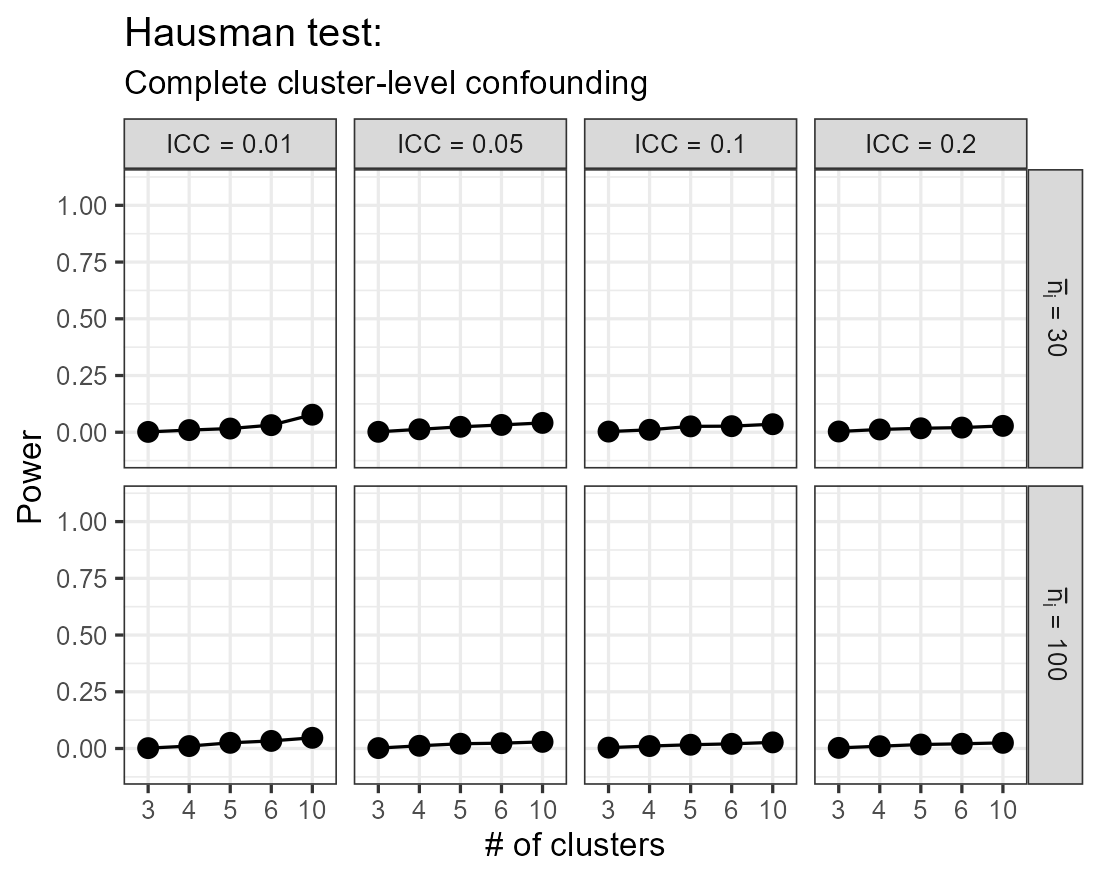


**References**

Gardiner, J. C., Luo, Z., & Roman, L. A. (2009). Fixed effects, random effects and GEE: What are the differences? *Statistics in Medicine*, *28*(2), 221–239. https://doi.org/10.1002/sim.3478

Hausman, J. A. (1978). Specification Tests in Econometrics. *Econometrica*, *46*(6), 1251–1271. https://doi.org/10.2307/1913827

Wooldridge, J. M. (2010). *Econometric Analysis of Cross Section and Panel Data* (2. ed). MIT Press.
